# Augmented Reality–Based Training for a Rapid Blood Transfusion Device Among Emergency Nurses: Randomized Controlled Trial

**DOI:** 10.2196/98073

**Published:** 2026-08-13

**Authors:** Wooyoung Jang, Wooree Chai, Yeji Kim, Youjung Moon, Suyoung Yoo, Sun Gyoung Na, Soyeon Shin, Meong Hi Son

**Affiliations:** 1 Department of Nursing, Samsung Medical Center, Seoul, Seoul, Republic of Korea; 2 Research Institute for Future Medicine, Samsung Medical Center, Seoul, Seoul, Republic of Korea; 3 Department of Emergency Medicine, Samsung Medical Center, 81, Irwon-ro, Gangnam-gu, Seoul, Seoul, 06351, Republic of Korea, 82 3410-2053

**Keywords:** augmented reality, nursing education, clinical competency, self-efficacy, educational satisfaction, rapid infusion, medical device training, randomized controlled trial

## Abstract

**Background:**

Emergency nurses must be proficient in operating the Level-1 rapid infusion system to manage hypovolemic shock effectively. However, training opportunities for this infrequently used but life-critical device remain scarce, owing to resource constraints and limited access to equipment. Augmented reality (AR) has emerged as a promising educational technology that provides immersive, hands-on learning experiences without compromising patient safety; yet its application to specialized medical device training in nursing has not been rigorously evaluated.

**Objective:**

This study aimed to evaluate the effects of an AR-based training program using Microsoft HoloLens 2 on emergency nurses’ clinical competency, self-efficacy, and educational satisfaction in operating the Level-1 rapid infusion system, compared with traditional guideline-based self-directed learning.

**Methods:**

A posttest-only randomized controlled trial was conducted at Samsung Medical Center in Seoul, Republic of Korea. Between July 17 and July 20, 2023, 42 registered nurses with no prior Level-1 experience were enrolled and randomly assigned in a 1:1 ratio to an experimental group receiving AR-based training on a single HoloLens 2 device (n=21) or a control group performing self-directed learning from a printed manual (n=21). Clinical competency was assessed by time (learning and performance), accuracy (a manufacturer-aligned checklist scored out of 100, and an expert-validated 22-step pass or fail evaluation), and the number of assistance requests. Self-efficacy (6-item scale; Cronbach α=0.80) and educational satisfaction (4-item scale; Cronbach α=0.87) were measured by questionnaire. Because most outcomes were non-normally distributed, groups were compared using the Mann-Whitney *U* test, with data reported as medians and IQRs.

**Results:**

Learning time was longer in the experimental group (median 18.20, IQR 15.48‐21.67 vs 8.98, IQR 5.85‐11.68 min; *P*<.001), but device setup time was markedly shorter (3.67, IQR 2.90‐4.63 vs 9.85, IQR 8.03‐11.35 min; *P*<.001). The experimental group achieved higher median device operation competency scores (90.00, IQR 80.00‐100.00 vs 70.00, IQR 50.00‐75.00 of 100; *P*<.001), passed more of the 22 evaluation steps (20.00, IQR 20.00‐22.00 vs 16.00, IQR 13.00‐18.00; *P*<.001), and required fewer assistance requests (0.00, IQR 0.00‐1.00 vs 2.00, IQR 2.00‐3.00; *P*<.001). Self-efficacy (20.00, IQR 18.00‐25.00 vs 16.50, IQR 13.00‐20.00; *P*=.003) and educational satisfaction (18.00, IQR 16.00‐18.00 vs 12.00, IQR 9.75‐15.00; *P*<.001) were also significantly higher in the experimental group. Effect sizes for the principal competency outcomes were large (Cohen *d*=1.2‐3.1).

**Conclusions:**

AR-based training significantly improved emergency nurses’ clinical competency, self-efficacy, and educational satisfaction in operating the Level-1 rapid infusion system compared with traditional self-directed learning. Despite requiring longer initial learning time, AR training produced faster device setup, greater accuracy, and enhanced learner independence. These findings suggest that AR technology can serve as an effective and scalable training solution for infrequently used but critically important medical devices in emergency care settings.

## Introduction

The rapid evolution of medical technology presents a persistent challenge for nursing education. New medical devices are introduced frequently, each requiring dedicated training for safe and effective operation [1,2]. Maintaining clinical competency across a growing inventory of devices is demanding, particularly for equipment that is infrequently used yet essential during emergencies [3,4]. Research consistently demonstrates that hands-on practice is the most effective approach for developing procedural proficiency [5]; however, providing individualized, device-specific training at scale remains logistically and financially prohibitive in most clinical settings.

Massive transfusion using the Level-1 rapid infusion system exemplifies this training dilemma. Although massive transfusion is performed infrequently, it is a lifesaving procedure required in urgent situations such as hypovolemic shock, severe trauma, and major surgical hemorrhage. Emergency nurses must be able to assemble, prime, and operate the Level-1 device swiftly and accurately under extreme time pressure. In practice, however, limited hands-on exposure leads to wide variability in skill levels and increased dependence on senior team members during critical resuscitation events, potentially compromising patient outcomes [6,7].

Augmented reality (AR) has emerged as a promising educational technology that addresses these constraints by overlaying digital instructional content onto the physical environment, allowing learners to practice with real equipment under guided conditions without risk to patients. A recent systematic mapping review of 156 studies published between 2010 and 2025 confirmed that AR is an effective educational tool in health sciences, improving hands-on skill acquisition, clinical competencies, and learner engagement while offering scalable, cost-effective solutions even in resource-limited settings [8]. In the specific context of medical device training, randomized trials have demonstrated that AR-based instruction for cardiopulmonary resuscitation and intubation significantly improves clinical performance compared with traditional methods [9]. Furthermore, AR supports the standardization of training by delivering consistent instructional content regardless of instructor expertise, which is particularly advantageous for devices that individual instructors may themselves rarely operate [10].

Despite this growing body of evidence, no prior study has specifically evaluated AR-based training for the Level-1 rapid infusion system. More broadly, the application of AR to training for infrequently used but critically important medical devices in nursing remains underexplored. Beyond individual skill development, AR-based training has implications for nursing management; standardized digital training programs can reduce dependence on instructor availability, ensure uniform skill acquisition across nursing teams, and support continuous professional development in resource-constrained environments [11-13]. Understanding whether AR can effectively address these dual objectives, enhancing individual competency while supporting organizational training goals, is essential for informed adoption decisions.

This study aimed to evaluate the effects of an AR-based training program using Microsoft HoloLens 2 on emergency nurses’ clinical competency, self-efficacy, and educational satisfaction in operating the Level-1 rapid infusion system. We hypothesized that AR-based training would produce superior outcomes across all 3 domains compared with traditional guideline-based self-directed learning.

## Methods

### Study Design

This study used a randomized controlled trial (RCT) with a posttest-only design.

### Participants and Randomization

Registered nurses working in the emergency department of Samsung Medical Center in Seoul, Republic of Korea, were recruited between July 17 and July 20, 2023. The required sample size of 42 participants (21 per group) was determined a priori using G*Power 3.1, based on an effect size (Cohen *d*) of 0.9, a significance level (α) of .05, and a statistical power (1−β) of .80.

Eligibility criteria included (1) voluntary participation with informed consent and (2) no prior experience operating the Level-1 device. Participants were randomly assigned to the experimental or control group through concealed allocation: each participant drew a slip of paper from an opaque box indicating group assignment in a 1:1 ratio. Both groups completed training and evaluation at the same location with 1:1 supervision.

### Interventions

#### AR-Based Training (Experimental Group)

**Figure 1.**
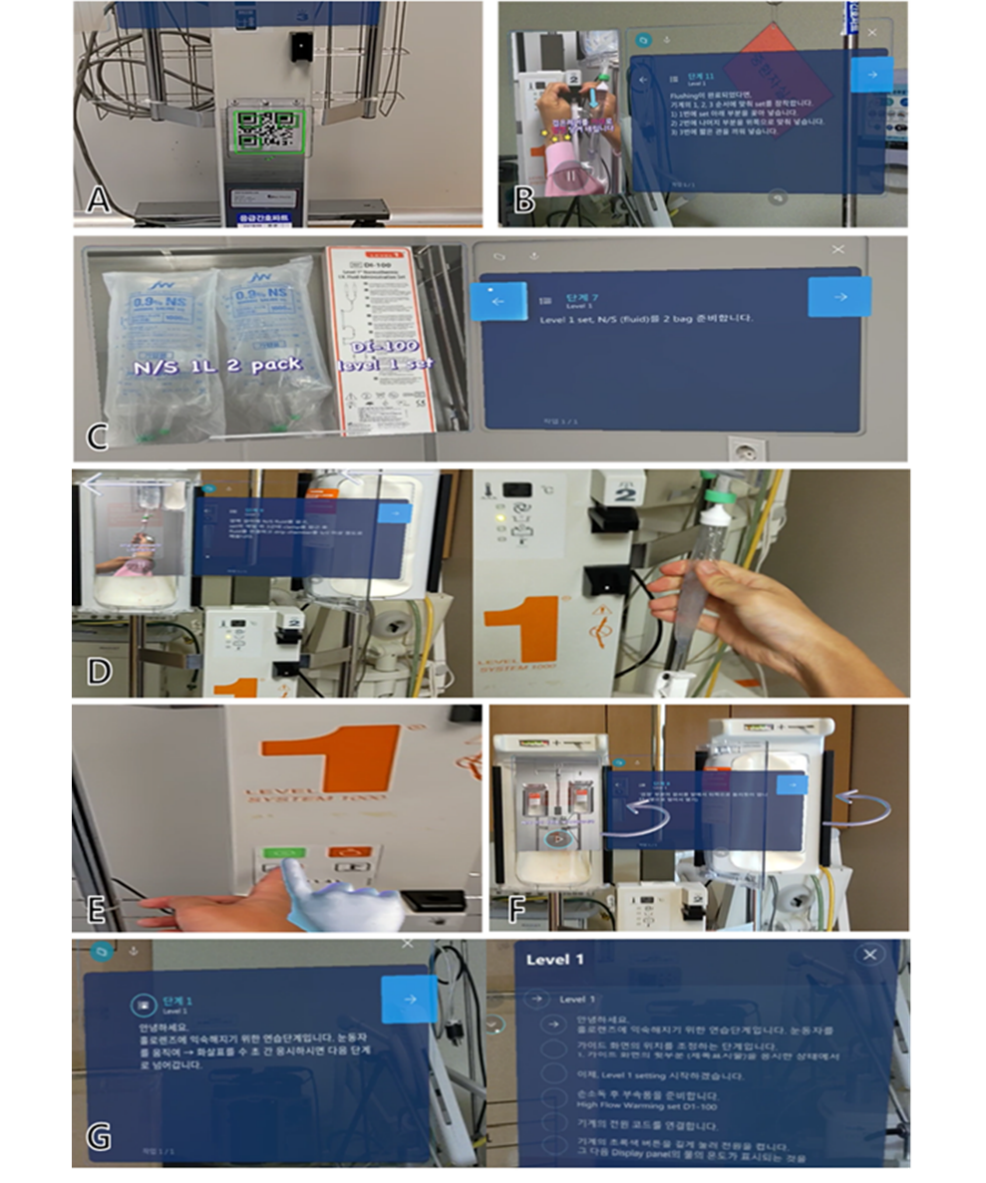
Trainee’s view during augmented reality–based training for the Level-1 rapid infusion system. (A) The anchor affixed to the device retrieves prestored instructional content and serves as a spatial reference for hologram positioning. (B) Finger-shaped holograms guide users to button locations. (C) Required materials for each step are displayed in advance. (D) Learning objectives appear in a blue instructional window. (E) Short instructional videos are played as holograms for complex operations. (F) The trainer’s hand movements are displayed for procedures that require visual demonstration. (G) The step navigation interface: a practice card that advances to the next step when the trainee gazes at the arrow (left) and the expanded step list for the Level-1 setup procedure (right).

The experimental group received AR-based training using Microsoft HoloLens 2, a head-mounted mixed reality device that projects interactive holographic content onto the user’s real-world field of view. The AR training module was developed using Microsoft Dynamics 365 Guides [14], an authoring application for creating step-by-step holographic work instructions, and was delivered on a single HoloLens 2 device. The training program guided participants through the Level-1 setup and operation process in a step-by-step sequence. Holographic overlays provided visual instructions, material checklists, finger-shaped pointing guides for button locations, and short procedural videos for complex steps (Figure 1). A physical anchor affixed to the device served as a spatial reference point for hologram positioning. This design allowed participants to interact directly with the actual equipment while receiving real-time digital guidance.

#### Traditional Training (Control Group)

The control group completed self-directed learning using the Level-1 nursing practice guidelines, a standardized printed manual developed by emergency nurses with more than 10 years of clinical experience. The manual describes the complete procedure from device power-on through system setup and rapid infusion, with step-by-step tasks and model answers. Participants studied the manual independently and then performed the procedure.

### Outcome Measures

#### Clinical Competency

Clinical competency was assessed across 3 domains. Time was measured as learning time (time to complete the educational content) and performance time, further segmented into device setup, fluid infusion, and blood transfusion times. Accuracy was evaluated using two instruments: (1) a manufacturer-aligned Level-1 checklist comprising 4 items scored out of 100 and (2) an expert-validated 22-step evaluation form derived from Level-1 nursing practice guidelines, with each step scored as pass or fail (Multimedia Appendix 1). Because the 2 accuracy instruments were intended to capture the same underlying construct, their convergent validity was examined using the Spearman rank correlation. Independence was measured by the number of assistance requests made during the performance evaluation.

#### Self-Efficacy

Self-efficacy was measured using a 6-item modified scale adapted from Compeau and Higgins [15] and Abdul Hamid et al [10], with 1 reverse-scored item on a 5-point Likert scale (total score range 6‐30; higher scores indicate greater self-efficacy). Expert validation yielded a content validity index of 1.0, and the Cronbach α was 0.80 in this study (Multimedia Appendix 2 [10,15,16]).

#### Educational Satisfaction

Educational satisfaction was assessed using 4 items on a 5-point Likert scale measuring confidence, perceived benefit, and willingness to recommend the training to colleagues (total score range 4‐20; higher scores indicate greater satisfaction) [16]. The Cronbach α was 0.87 in this study.

### Statistical Analysis

Data were analyzed using SPSS (version 18.0; IBM Corp). Normality was assessed using the Shapiro-Wilk test together with skewness values. Most continuous outcomes were nonnormally distributed, and the timed and count variables were markedly right-skewed (skewness ranging from +0.50 to +6.09). Accordingly, between-group differences in clinical competency, self-efficacy, and educational satisfaction were compared using the Mann-Whitney *U* test, and the corresponding data are summarized as medians with IQRs. Baseline homogeneity was assessed using the independent-samples *t* test. Convergent validity between the 2 accuracy instruments was evaluated using the Spearman rank correlation. All tests were 2-tailed, and statistical significance was set at *P*<.05.

### Ethical Considerations

This study was approved by the Samsung Medical Center Institutional Review Board (2023-06-084). All participants provided written informed consent prior to enrollment. Participation was voluntary, and participants could withdraw at any time without consequence. To ensure ethical fairness, participants in the control group were offered the opportunity to receive HoloLens-based training after the study was completed, if they wished. The clinical trial was registered at ClinicalTrials.gov (NCT06506851).

## Results

### Participant Characteristics

**Figure 2.**
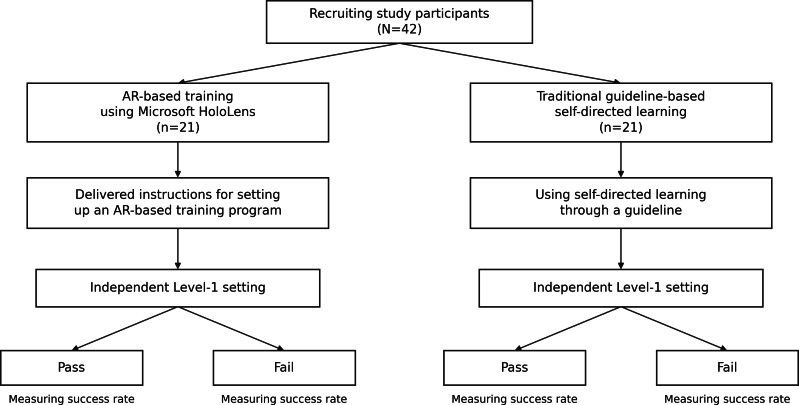
Flowchart of participant recruitment, randomization, and study procedures. AR: augmented reality.

A total of 42 participants (n=21, 50% per group) were enrolled and completed the study; there were no dropouts (Figure 2). The mean age was 28.00 (SD 3.93) years in the experimental group and 26.48 (SD 2.04) years in the control group. Clinical experience averaged 3.82 (SD 3.98) years and 2.32 (SD 2.17) years, respectively, with emergency department experience of 1.68 (SD 1.23) years and 1.75 (SD 1.09) years, respectively. No significant differences were found between groups in age, clinical experience, emergency department experience, or sex distribution, confirming baseline homogeneity (Table 1). One control-group participant did not complete the posttraining self-report questionnaire; therefore, the self-efficacy and educational satisfaction analyses include 20 control participants (Table 2), whereas all clinical competency analyses include the full sample (N=42).

**Table 1.** Baseline characteristics of participants.^a^

Characteristics	Experimental (n=21)	Control (n=21)	*t* test (*df*)^a^	*P* value
Age (y), mean (SD)	28.00 (3.93)	26.48 (2.04)	−1.575 (40)	.12
Clinical experience (y), mean (SD)	3.82 (3.98)	2.32 (2.17)	−1.514 (40)	.14
Emergency department experience (y), mean (SD)	1.68 (1.23)	1.75 (1.09)	0.198 (40)	.84
Sex (male), n (%)	3 (14)	5 (24)	−0.773 (40)	.44

a All between-group comparisons were made using the independent-samples *t* test.

**Table 2.** Self-efficacy scores^a^.

Items	Experimental (n=21), median (IQR)	Control (n=20)^b^, median (IQR)	Mann-Whitney *U* test	*P* value
Confident advising others	4.00 (3.00‐5.00)	2.00 (1.75‐3.00)	345.0	<.001
Confident troubleshooting problems	3.00 (3.00‐4.00)	2.00 (1.00‐3.00)	329.0	.001
Confident learning advanced skills	4.00 (3.00‐4.00)	3.00 (3.00‐4.00)	304.5	.008
Confident understanding terminology	3.00 (3.00‐4.00)	3.00 (2.75‐3.00)	256.0	.21
Confident describing functions	4.00 (3.00‐4.00)	3.00 (2.00‐3.00)	309.0	.007
Pressured when observed (reverse scored)	3.00 (2.00‐4.00)	3.00 (2.00‐3.25)	220.5	.80
Total	20.00 (18.00‐25.00)	16.50 (13.00‐20.00)	322.5	.003

a Data are presented as median (IQR); between-group comparisons used the Mann-Whitney *U* test.

b One control participant did not complete the posttraining questionnaire.

### Clinical Competency

Table 3 presents the clinical competency outcomes. Learning time was significantly longer in the experimental group (median 18.20, IQR 15.48‐21.67 min vs median 8.98, IQR 5.85‐11.68 min; Mann-Whitney *U*=377.0; *P*<.001), reflecting the more structured, step-by-step nature of the AR-guided program. However, this additional learning time translated into substantially faster performance: device setup time was markedly shorter in the experimental group (median 3.67, IQR 2.90‐4.63 min vs median 9.85, IQR 8.03‐11.35 min; Mann-Whitney *U*=3.5; *P*<.001), and total performance time was likewise shorter (median 5.83, IQR 4.80‐6.68 min vs median 13.63, IQR 10.33‐15.17 min; Mann-Whitney *U*=26.0; *P*<.001). Of note, the between-group difference in total performance time was not detected by a parametric test (independent-samples *t* test: *P*=.06) but was highly significant under the Mann-Whitney *U* test, underscoring the importance of using distribution-appropriate methods for these skewed data.

**Table 3.** Clinical competency outcomes^a^.

Variables	Experimental (n=21), median (IQR)	Control (n=21), median (IQR)	Mann-Whitney *U* test	*P* value
Learning time (min)	18.20 (15.48‐21.67)	8.98 (5.85‐11.68)	377.0	<.001
Total performance time (min)	5.83 (4.80‐6.68)	13.63 (10.33‐15.17)	26.0	<.001
Device setup time (min)	3.67 (2.90‐4.63)	9.85 (8.03‐11.35)	3.5	<.001
Fluid infusion time (min)	0.50 (0.37‐0.62)	0.65 (0.33‐0.92)	188.5	.43
Blood transfusion time (min)	1.50 (1.30‐1.85)	2.05 (1.37‐2.48)	147.0	.08
Device operation competency score	90.00 (80.00‐100.00)	70.00 (50.00‐75.00)	407.0	<.001
Checklist completion (22 items passed)	20.00 (20.00‐22.00)	16.00 (13.00‐18.00)	414.0	<.001
Device setup tasks (15 items)	14.00 (13.00‐15.00)	10.00 (9.00‐12.00)	410.5	<.001
Fluid infusion tasks (2 items)	2.00 (2.00‐2.00)	2.00 (2.00‐2.00)	252.5	.17
Blood transfusion tasks (5 items)	5.00 (5.00‐5.00)	3.00 (2.00‐5.00)	309.5	.02
Assistance requests, n	0.00 (0.00‐1.00)	2.00 (2.00‐3.00)	14.0	<.001

a Data are presented as median (IQR); between-group comparisons used the Mann-Whitney *U* test.

**Figure 3.**
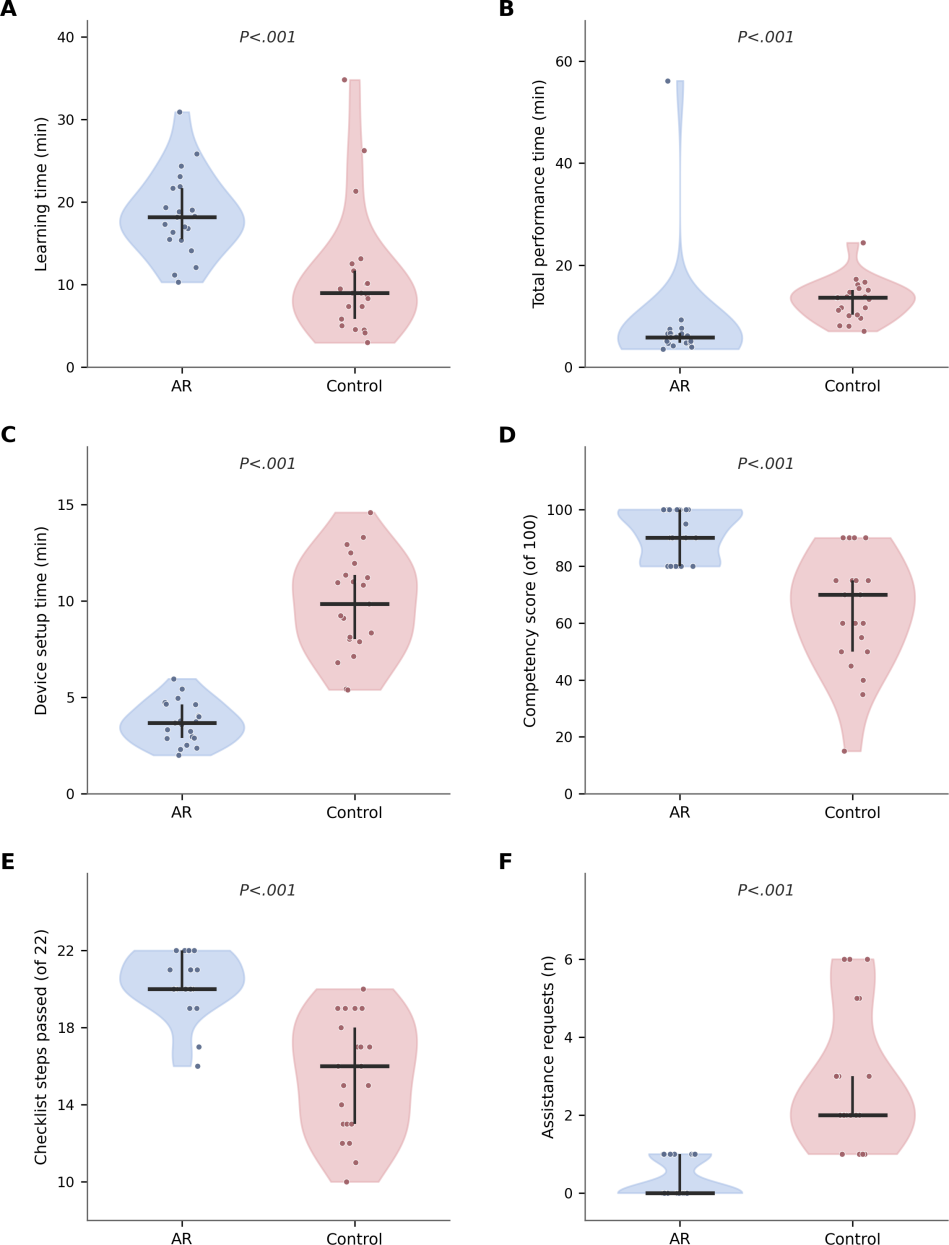
Distribution of the principal clinical competency outcomes by group (N=42). (A) Learning time, (B) total performance time, (C) device setup time, (D) device operation competency score, (E) checklist steps passed of 22, and (F) number of assistance requests. Violin plots show the kernel density of each outcome; overlaid points represent individual participants, and horizontal bars denote medians with IQRs. *P* values are from the Mann-Whitney *U* test. AR: augmented reality.

The experimental group also demonstrated superior accuracy, with a median device operation competency score of 90.00 (IQR 80.00‐100.00) compared with a median of 70.00 (IQR 50.00‐75.00) in the control group (Mann-Whitney *U*=407.0; *P*<.001). On the 22-step checklist, the experimental group passed a median of 20.00 (IQR 20.00‐22.00) steps compared with a median of 16.00 (IQR 13.00‐18.00) steps in the control group (Mann-Whitney *U*=414.0; *P*<.001; Multimedia Appendix 3). The 2 accuracy instruments were strongly correlated (Spearman ρ=0.86; *P*<.001), supporting their convergent validity. Notably, the experimental group required significantly fewer assistance requests (median 0.00, IQR 0.00‐1.00 vs median 2.00, IQR 2.00‐3.00; Mann-Whitney *U*=14.0; *P*<.001), indicating that AR training fostered greater independence during device operation. The distributions of the principal competency outcomes are shown in Figure 3.

### Self-Efficacy

The experimental group reported significantly higher total self-efficacy scores (median 20.00, IQR 18.00‐25.00) than the control group (median 16.50, IQR 13.00‐20.00; Mann-Whitney *U*=322.5; *P*=.003). The largest between-group differences were observed in confidence in advising others about Level-1 operation (Mann-Whitney *U*=345.0; *P*<.001) and confidence in troubleshooting device problems (Mann-Whitney *U*=329.0; *P*=.001). The reverse-scored item measuring performance pressure when observed by others showed no significant difference between groups (*P*=.80; Table 2).

### Educational Satisfaction

Educational satisfaction was significantly higher in the experimental group across all items (Table 4). The total score was higher in the experimental group (median 18.00, IQR 16.00‐18.00) than in the control group (median 12.00, IQR 9.75‐15.00; Mann-Whitney *U*=385.0; *P*<.001). Participants in the experimental group reported significantly greater confidence in performing rapid Level-1 setups independently (median 4.00, IQR 3.00‐4.00 vs median 2.00, IQR 1.75‐3.00; Mann-Whitney *U*=369.5; *P*<.001) and higher perceived benefit from the training (median 5.00, IQR 5.00‐5.00 vs median 4.00, IQR 3.00‐4.00; Mann-Whitney *U*=354.0; *P*<.001).

**Table 4.** Educational satisfaction scores^a^.

Items	Experimental (n=21), median (IQR)	Control (n=20)^b^, median (IQR)	Mann-Whitney *U* test	*P* value
Confidence in Level-1 setup	4.00 (3.00‐4.00)	2.00 (1.75‐3.00)	369.5	<.001
Confidence in rapid infusion	4.00 (3.00‐4.00)	3.00 (1.75‐3.25)	326.0	.002
Perceived benefit from training	5.00 (5.00‐5.00)	4.00 (3.00‐4.00)	354.0	<.001
Willingness to recommend training	5.00 (5.00‐5.00)	3.50 (2.00‐5.00)	343.0	<.001
Total	18.00 (16.00‐18.00)	12.00 (9.75‐15.00)	385.0	<.001

a Data are presented as median (IQR); between-group comparisons used the Mann-Whitney *U* test.

b One control participant did not complete the posttraining questionnaire.

## Discussion

### Principal Findings

This study provides the first randomized controlled evidence that AR-based training significantly improves emergency nurses’ clinical competency, self-efficacy, and educational satisfaction in operating the Level-1 rapid infusion system compared with traditional guideline-based self-directed learning. The experimental group showed a 62% reduction in median device setup time (3.67, IQR 2.90-4.63 min vs 9.85, IQR 8.03-11.35 min), a 20-point higher median device operation competency score (90 vs 70 out of 100), and a reduction in the median number of assistance requests from 2 to 0. These results suggest that AR technology can effectively address a critical gap in nursing education: the challenge of training for infrequently used but life-critical medical devices.

### Comparison With Prior Work

Our findings align with and extend the broader evidence base supporting AR in health professional education. A systematic mapping review of 156 studies (2010‐2025) confirmed that AR effectively supports hands-on skill acquisition and clinical competency development across health sciences disciplines [8], and an umbrella review of virtual reality (VR) and AR in medical education similarly concluded that these technologies enhance specific professional competencies compared with traditional methods [17]. In the nursing domain specifically, a 2025 scoping review of 41 AR studies in nursing found that head-mounted displays were the most commonly used AR device type, with more than 90% of studies published since 2020 and more than half using RCT designs [18,19]. Recent randomized trials have demonstrated that AR-based training improves procedural performance in specific clinical tasks: Sun et al [5] showed that an AR app significantly improved nurses’ advanced cardiac life support knowledge and crash cart skills, and Othman et al [13] reported that combining AR with gamification significantly enhanced critical care nursing students’ self-efficacy and motivation. A 2025 RCT of VR and AR in emergency health care training further confirmed that immersive technologies improve emergency skill acquisition compared with traditional instruction [20]. Our study adds to this literature by demonstrating AR’s efficacy for a specialized emergency medical device, the Level-1 rapid infuser, which poses unique training challenges owing to its infrequent use and high-stakes application context.

The effect sizes observed in this study were notably large. Expressed as Cohen *d*, the between-group differences were approximately 3.1 for device setup time, 2.0 for both the 22-step checklist and the number of assistance requests, 1.8 for the competency score, and 1.2 for learning time, substantially larger than the moderate-to-large effects typically reported in AR or immersive nursing education trials, which generally fall in the range of Cohen *d*≈0.5 to 1.0 (eg, the improvements in knowledge, skills, self-efficacy, and motivation reported by Sun et al [5] and Othman et al [13]). Several design factors most plausibly account for this magnitude. First, the comparator was self-directed learning from a printed manual without any hands-on guidance, a deliberately conservative, real-world control that maximizes the contrast with real-time, in situ AR guidance. Second, all participants were novices with no prior Level-1 experience, leaving substantial room for improvement and limiting ceiling effects in the AR group. Third, the AR program delivered step-by-step holographic instruction anchored to the actual device (finger-pointing cues, material checklists, and embedded video), tightly coupling instruction to the physical task and reducing cognitive load during a procedurally complex, low-frequency procedure. Fourth, the single-device focus permitted concentrated, uninterrupted practice. These instructional design features, rather than the technology alone, are the most likely drivers of the large effects, and the magnitude should therefore be interpreted in the context of this conservative comparator. Cohen *d* is reported here for comparability with the exemplar studies; the corresponding nonparametric effect sizes (rank-biserial correlation) were of similar magnitude (*r*=0.71 to 0.98).

The finding that AR training required significantly more learning time yet produced faster performance is consistent with prior work on immersive training modalities [21]. This pattern suggests that AR’s step-by-step, interactive format promotes deeper encoding of procedural knowledge during the learning phase, which subsequently translates into more efficient and accurate performance. A similar trade-off was observed in a controlled trial of AR-assisted intubation training, in which the AR group took longer to complete the training but demonstrated greater adherence to evidence-based procedures [22]. A systematic review and meta-analysis of immersive technology–based nursing education using the GRADE (Grading of Recommendations, Assessment, Development, and Evaluation) approach further supports that immersive technologies improve practical skill outcomes, although with varying effect sizes depending on the specific clinical domain [3].

The significant improvement in self-efficacy, particularly in confidence-related domains such as advising colleagues and troubleshooting equipment, is consistent with findings from studies showing that immersive learning environments foster greater confidence through realistic practice opportunities [21,23]. Notably, there was no significant between-group difference in the reverse-scored item measuring performance anxiety, suggesting that AR training enhanced positive confidence without increasing stress. A recent review of the HoloLens platform for health care simulation confirmed that mixed reality training supports competency development across diverse medical and procedural domains, with learners consistently reporting increased confidence [24]. The substantial improvement in educational satisfaction echoes findings from our prior mixed methods study evaluating AR adoption for nurses in the intensive care unit [4], reinforcing the acceptability and user experience advantages of AR-based educational approaches.

### Implications for Nursing Education and Management

These findings carry practical implications for nursing management. The Level-1 rapid infuser represents a category of medical devices that are critical during emergencies but used too infrequently for conventional training programs to maintain competency effectively. AR-based training offers a scalable solution: once developed, the digital content can be deployed across multiple sites with consistent quality, reducing dependence on expert instructors and physical training resources [11,25,26]. The significant reduction in assistance requests observed in the experimental group suggests that AR training may improve team efficiency during actual resuscitation events by reducing the need for on-the-job guidance from senior staff. For nursing managers seeking to implement digital transformation initiatives [13,27,28], AR-based device training represents a concrete, evidence-supported application with measurable outcomes.

### Limitations

This study has several limitations that should be considered when interpreting the results. First, outcomes were assessed immediately after the intervention, and the long-term retention of skills and knowledge was not evaluated. Future studies should incorporate follow-up assessments at defined intervals (eg, 1, 3, and 6 months) to determine the durability of training effects and the need for refresher training. Second, this was a single-center study conducted within one emergency department, which limits the generalizability of the findings. Multicenter trials involving nurses from diverse clinical settings, including intensive care units and surgical departments, would provide a broader understanding of AR training’s applicability. Third, the study assessed individual performance metrics only; team-based outcomes such as communication, coordination, and collective decision-making during simulated resuscitation scenarios were not evaluated. Fourth, blinding was not feasible owing to the nature of the intervention, introducing the possibility of performance bias. Finally, cost-effectiveness was not assessed; future research should analyze the financial feasibility of AR training programs, including equipment acquisition, content development, and maintenance costs relative to improved clinical outcomes.

### Conclusions

This RCT demonstrates that AR-based training using Microsoft HoloLens 2 significantly enhances emergency nurses’ clinical competency, self-efficacy, and educational satisfaction in operating the Level-1 rapid infusion system. Despite requiring additional initial learning time, AR training produced faster, more accurate, and more independent device operation compared with traditional self-directed learning. These findings position AR technology as a viable and effective educational tool for medical devices that are infrequently used but critically important in emergency care. Future research should evaluate long-term skill retention, cost-effectiveness, and the applicability of AR-based training across a broader range of medical devices and clinical settings.

## Supplementary material

10.2196/98073 Multimedia Appendix 1 The 22-step clinical competency evaluation form for Level-1 rapid infusion system operation.

10.2196/98073 Multimedia Appendix 2 Self-efficacy and Educational Satisfaction Scale items with scoring instructions.

10.2196/98073 Multimedia Appendix 3 Item-level pass rate analysis of the 22-step evaluation by procedural phase and group.

10.2196/98073 Checklist 1 CONSORT (Consolidated Standards of Reporting Trials) checklist.

## Abbreviations

AR: augmented reality
GRADE: Grading of Recommendations, Assessment, Development, and Evaluation
RCT: randomized controlled trial
VR: virtual reality
