# Supplementary material for: Augmented Reality–Based Training for a Rapid Blood Transfusion Device Among Emergency Nurses: Randomized Controlled Trial
Source: JMIR Med Educ. 2026 Aug 13;12:e98073. doi: 10.2196/98073 (PMC13472624; doi:10.2196/98073)
Supplement: Supplementary file 1 [file mededu-v12-e98073-s004.pdf]

CONSORT-EHEALTH Checklist (V 1.6.1) - Filled

Manuscript #98073: Augmented Reality-Based Training for a Rapid Blood Transfusion Device Among Emergency Nurses: Randomized Controlled Trial

Citation: Eysenbach G, CONSORT-EHEALTH Group. CONSORT-EHEALTH: Improving and Standardizing Evaluation Reports of Web-based and Mobile Health Interventions. J Med Internet Res 2011;13(4):e126. doi:10.2196/jmir.1923. PMID:22209829

Item	CONSORT-EHEALTH Item	Manuscript Quote/Response	Location in Manuscript
**Title and Abstract**			
1a	Identification as RCT in title	"Augmented Reality-Based Training for a Rapid Blood Transfusion Device Among Emergency Nurses: Randomized Controlled Trial"	Title
1a-i	Identification as eHealth trial	AR-based training using Microsoft HoloLens 2 is identified as the eHealth intervention in the title and abstract.	Title, Abstract
1b	Structured abstract	Structured abstract with Background, Objective, Methods, Results, Conclusions, Trial Registration, and Keywords.	Abstract
**Introduction**			
2a	Scientific background and rationale	Background on Level-1 rapid infuser training challenges, AR in nursing education, and the specific gap addressed.	Introduction, paras 1-3
2a-i	Problem and type of system/solution	"AR has emerged as a promising educational technology that overlays digital instructional content onto the physical environment, allowing learners to practice with real equipment under guided conditions."	Introduction, para 3

Item	CONSORT-EHEALTH Item	Manuscript Quote/Response	Location in Manuscript
2a-ii	Literature review of similar systems	Systematic mapping review of 156 AR studies [21], scoping review of 41 AR studies in nursing [22], RCTs in resuscitation [3,10], gamification [15], and HoloLens review [25] cited.	Introduction, para 3
2b	Specific objectives/hypotheses	“We hypothesized that AR-based training would produce (1) faster device setup time and higher procedural accuracy (clinical competency), (2) greater confidence in independent device operation (self-efficacy), and (3) higher perceived educational value (satisfaction).”	Introduction, para 5
**Methods**			
3a	Trial design	“This study used a randomized controlled trial with a posttest-only design.”	Methods: Study Design
3b	Important changes after commencement	No changes to methods after trial commencement.	N/A - no changes
4a	Eligibility criteria	”(1) voluntary participation with informed consent and (2) no prior experience operating the Level-1 device.” Emergency department RNs at Samsung Medical Center.	Methods: Participants
4a-i	Mention of IRB approval	“Approved by the Institutional Review Board of	Methods: Ethical Considerations

Item	CONSORT-EHEALTH Item	Manuscript Quote/Response	Location in Manuscript
		Samsung Medical Center (IRB No. 2023-06-084)."	
4a-ii	Informed consent	"All participants provided written informed consent after receiving a detailed explanation of the study's purpose and procedures."	Methods: Study Design
4a-iii	Conflict of interest	"None declared."	Conflicts of Interest
4b	Settings and locations	Emergency department, Samsung Medical Center, Seoul, Republic of Korea. Training and evaluation at the same location.	Methods: Participants
**Interventions**			
5	Interventions for each group	AR group: HoloLens 2 with Dynamics 365 Guides, step-by-step holographic overlays. Control group: printed Level-1 nursing practice guidelines for self-directed learning.	Methods: Interventions
5-i	Description of steps/key features	"The training program guided participants through the Level-1 setup and operation process in a step-by-step sequence. Holographic overlays provided visual instructions, material checklists, finger-shaped pointing guides for button locations, and short procedural videos for complex steps."	Methods: AR-Based Training

Item	CONSORT-EHEALTH Item	Manuscript Quote/Response	Location in Manuscript
5-ii	Describe interactivity components	Participants interact directly with actual Level-1 equipment while receiving real-time holographic guidance. Physical anchor affixed to device for spatial hologram positioning.	Methods: AR-Based Training
5-iii	Describe use of multimedia	Short instructional videos played as holograms for complex operations; trainer's hand movements displayed for procedures requiring visual demonstration (Figure 1).	Methods: AR-Based Training, Figure 1
5-iv	Level of human involvement	1:1 supervision during training. A research team member (KM Min) provided HoloLens technical support. No instructor-led procedural guidance during AR training.	Methods: AR Program Development
5-v	Describe automated functions	Dynamics 365 Guides delivers automated step-by-step holographic instructions without instructor intervention. No adaptive/tailoring features.	Methods: Interventions
5-vi	Describe incentives	"Participants received a gift card (approximately US $7) as compensation for their time."	Methods: Participants
**Outcomes**			
6a	Primary and secondary outcomes	Primary: clinical competency (time, accuracy, independence). Secondary:	Methods: Outcome Measures

Item	CONSORT-EHEALTH Item	Manuscript Quote/Response	Location in Manuscript
		self-efficacy (6-item scale), educational satisfaction (4-item scale).	
6a-i	Online questionnaires validation	Self-efficacy modified from Compeau & Higgins [4]; CVI = 1.0 validated by 3 domain experts; Cronbach α = .80. Paper-based administration, not online.	Methods: Self-efficacy
6a-ii	Use/dosage definition	Single training session per participant. Learning time measured from start to self-reported completion.	Methods: Outcome Measures
6b	Changes to outcomes after commencement	No changes to outcome measures after trial commencement.	N/A
**Sample Size**			
7a	Sample size determination	"G*Power 3.1: independent two-tailed t-test, α = .05, power = 0.80, d = 0.9, yielding n = 21 per group (N = 42)."	Methods: Participants
7a-i	Expected attrition	No attrition expected or observed (posttest-only, single-session design). All 42 participants completed the study.	Methods/Results
7b	Interim analyses	No interim analyses or stopping guidelines were applied.	N/A
**Randomization**			

Item	CONSORT-EHEALTH Item	Manuscript Quote/Response	Location in Manuscript
8a	Sequence generation method	Simple randomization using opaque sealed envelopes prepared by a researcher not involved in data collection.	Methods: Participants
8b	Type of randomization	Simple randomization, 1:1 ratio, using sealed envelopes.	Methods: Participants
9	Allocation concealment	"Participants drew envelopes sequentially upon enrollment, and allocation was revealed only after informed consent was obtained and baseline demographics were collected."	Methods: Participants
10	Implementation	A researcher not involved in data collection prepared envelopes. Participants drew envelopes sequentially.	Methods: Participants
**Blinding**			
11a	Who was blinded	"Blinding of participants and outcome assessors was not feasible owing to the nature of the AR intervention. The assessors were aware of group allocation." Acknowledged as limitation.	Methods/Limitations
11b	Description of similarity of interventions	Not applicable; interventions were inherently different (AR headset vs printed manual).	N/A
**Statistical Methods**			

Item	CONSORT-EHEALTH Item	Manuscript Quote/Response	Location in Manuscript
12a	Statistical methods	“Normality assessed using Shapiro-Wilk test. Non-normal: Mann-Whitney U. Normal: independent t-test. Cohen d and 95% CIs reported. Bonferroni correction applied (α = .005 for 10 primary comparisons).”	Methods: Statistical Analysis
12a-i	Imputation techniques for attrition	No attrition; all 42 participants completed all measures. No imputation needed.	Results
12b	Subgroup analyses	No subgroup analyses performed.	N/A
**Results - Participant Flow**			
13a	Participant flow diagram (CONSORT)	Figure 2: CONSORT flow diagram showing enrollment (n=42), allocation (n=21 per group), no losses, and analysis (n=21 per group).	Results, Figure 2
13b	Losses and exclusions	No losses to follow-up, no exclusions after randomization, no protocol deviations.	Results
**Results - Recruitment and Baseline**			
14a	Dates of recruitment and follow-up	“Recruited between July 17 and July 20, 2023.” Posttest-only; no follow-up period.	Methods/Results

Item	CONSORT-EHEALTH Item	Manuscript Quote/Response	Location in Manuscript
14b	Why trial ended	Trial ended when target sample size (N=42) was reached.	N/A
15	Baseline demographics table	Table 1: Age, clinical experience, ED experience, sex by group with mean differences and 95% CIs.	Results, Table 1
16	Numbers analyzed	All 42 participants (21 per group) included in analysis. No intention-to-treat issues (no dropouts).	Results
**Results - Outcomes and Estimation**			
17a	Outcomes and estimation	Tables 2-4 present all outcomes with means, SDs, test statistics, P values, Cohen d, and 95% CIs.	Results, Tables 2-4
17a-i	Basis for outcomes	Outcomes based on single posttest assessment. No subgroup differences explored.	Results
17b	Binary outcomes	22-step checklist items scored as pass/fail (binary). Reported as mean steps passed.	Results, Table 2
18	Ancillary analyses	Item-level analysis of 22-step checklist provided in Multimedia Appendix 4.	Multimedia Appendix 4

Item	CONSORT-EHEALTH Item	Manuscript Quote/Response	Location in Manuscript
19	Harms	No adverse events reported. No participants reported discomfort with the HoloLens device.	Results/Discussion
19-i	Unintended consequences	No unintended consequences of the intervention were observed.	N/A
**Discussion**			
20	Trial limitations	Eight limitations discussed: (1) no long-term follow-up, (2) single center, (3) no baseline assessment, (4) no blinding, (5) no inter-rater reliability, (6) young age bias, (7) unmeasured AR/VR experience, (8) no cost-effectiveness.	Discussion: Limitations
20-i	Discuss generalizability to other populations	"The relatively young mean age of participants (27.2 years) and their likely familiarity with digital technologies may limit generalizability to older nurses or those less experienced with technology."	Discussion: Limitations
20-ii	Discuss generalizability to other platforms	"The training content developed using Dynamics 365 Guides is platform-transferable and can be migrated to successor MR devices." HoloLens 2 discontinuation acknowledged.	Discussion

Item	CONSORT-EHEALTH Item	Manuscript Quote/Response	Location in Manuscript
21	Interpretation	AR training significantly improved all primary outcomes with large effect sizes. Results maintained after non-parametric re-analysis and Bonferroni correction.	Discussion: Principal Findings
22	Registration number	ClinicalTrials.gov NCT06506851	Abstract
23	Full trial protocol access	Study protocol available through ClinicalTrials.gov registration. IRB approval number: 2023-06-084.	Methods/Abstract
**Other Information**			
24	Funding sources	“Supported by the research fund of the Department of Nursing, Samsung Medical Center (No. SMC-NSD-2023-02) and a Korea Health Technology R&D Project grant (HI23C1234).”	Funding
25	Data access	Data available from corresponding author upon reasonable request.	N/A

This checklist was completed on the basis of the revised manuscript submitted in response to reviewer and editor comments.
