# Supplementary material for: Augmented Reality–Based Training for a Rapid Blood Transfusion Device Among Emergency Nurses: Randomized Controlled Trial
Source: JMIR Med Educ. 2026 Aug 13;12:e98073. doi: 10.2196/98073 (PMC13472624; doi:10.2196/98073)
Supplement: Supplementary file 2 [file mededu-v12-e98073-s001.docx]

**Multimedia Appendix 3**

**22-Step Clinical Competency Evaluation Form**

Derived from the Level-1® manufacturer's operational manual and the institution's nursing practice guidelines. Each step scored as Pass (1) or Fail (0). Validated by a panel of 3 domain experts (2 emergency nursing unit managers, 1 emergency department nurse with >10 years experience; I-CVI = 1.0 for all items).

| **Step** | **Procedure** | **Phase** |
| --- | --- | --- |
| 1 | Power on the Level-1® device | Setup |
| 2 | Check the device self-test completion | Setup |
| 3 | Open the disposable set packaging | Setup |
| 4 | Install the heat exchanger cartridge | Setup |
| 5 | Connect the infusion line to the heat exchanger | Setup |
| 6 | Secure the drip chamber in the correct position | Setup |
| 7 | Connect the pressure infusion bag | Setup |
| 8 | Hang the IV fluid bag on the IV pole | Setup |
| 9 | Prime the infusion line with IV fluid | Setup |
| 10 | Remove all air bubbles from the line | Setup |
| 11 | Set the target temperature on the device | Setup |
| 12 | Connect the patient line to the infusion set | Setup |
| 13 | Verify all connections are secure and leak-free | Setup |
| 14 | Confirm device readiness indicators | Setup |
| 15 | Set the infusion rate as prescribed | Setup |
| 16 | Initiate fluid infusion and confirm flow | Fluid Infusion |
| 17 | Monitor fluid level and device operation | Fluid Infusion |
| 18 | Verify blood product identification (patient ID matching) | Blood Transfusion |
| 19 | Connect blood product bag to the infusion line | Blood Transfusion |
| 20 | Adjust infusion rate for blood product administration | Blood Transfusion |
| 21 | Monitor for transfusion reactions during initial infusion | Blood Transfusion |
| 22 | Document transfusion initiation time and vital signs | Blood Transfusion |

*Note: Step descriptions are representative of the evaluation criteria. The actual clinical evaluation form included specific performance standards and observable behaviors for each step.*
