# Supplementary material for: Augmented Reality–Based Training for a Rapid Blood Transfusion Device Among Emergency Nurses: Randomized Controlled Trial
Source: JMIR Med Educ. 2026 Aug 13;12:e98073. doi: 10.2196/98073 (PMC13472624; doi:10.2196/98073)
Supplement: Supplementary file 3 [file mededu-v12-e98073-s002.docx]

**Multimedia Appendix 2**

**Self-Efficacy and Educational Satisfaction Scale Items**

**Self-Efficacy Scale**

Adapted from Compeau and Higgins [4] and Abdul Hamid et al [1]. Each item rated on a 5-point Likert scale (1 = strongly disagree, 2 = disagree, 3 = neutral, 4 = agree, 5 = strongly agree). Item 6 is reverse-scored: higher raw scores indicate greater perceived pressure (negative), so scores are reversed (6 − raw score) when computing the total. Total score range: 6–30 (higher = greater self-efficacy). Cronbach α = .80.

| **No.** | **Item** | **Direction** | **Scoring** |
| --- | --- | --- | --- |
| 1 | I could confidently advise others on how to operate the Level-1® device. | Positive | 1–5 |
| 2 | I could confidently troubleshoot problems that arise during Level-1® operation. | Positive | 1–5 |
| 3 | I could learn advanced skills related to Level-1® operation. | Positive | 1–5 |
| 4 | I could understand technical terminology related to the Level-1® device. | Positive | 1–5 |
| 5 | I could describe the functions and procedures of the Level-1® device to others. | Positive | 1–5 |
| 6 | I feel pressured when others are watching me operate the Level-1® device. | Reverse | 5–1* |

**Item 6 reverse-scored: raw score subtracted from 6 for total calculation.*

**Educational Satisfaction Scale**

Each item rated on a 5-point Likert scale (1 = strongly disagree to 5 = strongly agree). Total score range: 4–20 (higher = greater satisfaction). Adapted from Heo et al [7]. Cronbach α = .87.

| **No.** | **Item** | **Scoring** |
| --- | --- | --- |
| 1 | I feel confident that I can independently set up the Level-1® device quickly. | 1–5 |
| 2 | I feel confident that I can perform rapid infusion using the Level-1® device. | 1–5 |
| 3 | I found this training method helpful for learning Level-1® operation. | 1–5 |
| 4 | I would recommend this training method to my colleagues. | 1–5 |
