# Supplementary material for: Augmented Reality–Based Training for a Rapid Blood Transfusion Device Among Emergency Nurses: Randomized Controlled Trial
Source: JMIR Med Educ. 2026 Aug 13;12:e98073. doi: 10.2196/98073 (PMC13472624; doi:10.2196/98073)
Supplement: Supplementary file 4 [file mededu-v12-e98073-s003.docx]

**Multimedia Appendix 4**

**Item-Level Pass Rate Analysis by Group**

Pass rates are expressed as the mean number of steps passed per phase, with the percentage of the total steps in that phase.

| **Phase** | **Steps** | **AR Group (n=21)** | **Control (n=21)** | **Difference** |
| --- | --- | --- | --- | --- |
| Device Setup | 15 | 14.05 (93.7%) | 10.38 (69.2%) | +24.5 pp |
| Fluid Infusion | 2 | 1.95 (97.6%) | 1.76 (88.1%) | +9.5 pp |
| Blood Transfusion | 5 | 4.33 (86.7%) | 3.33 (66.7%) | +20.0 pp |
| **Total** | **22** | **20.33 (92.4%)** | **15.48 (70.3%)** | **+22.1 pp** |

*pp = percentage points.*

The largest performance gap was observed in the Device Setup phase (+24.5 percentage points), which encompasses the most complex multi-step assembly procedures. This finding is consistent with the hypothesis that AR-based holographic step-by-step guidance is most beneficial for procedurally complex tasks requiring precise sequential execution.

Fluid Infusion showed the smallest gap (+9.5 percentage points), likely reflecting the relative simplicity of this phase (2 steps) and the high baseline pass rate in both groups (>88%).
